# Correction: Comprehensive histochemical profiles of histone modification in male germline cells during meiosis and spermiogenesis: Comparison of young and aged testes in mice

**DOI:** 10.1371/journal.pone.0237446

**Published:** 2020-10-28

**Authors:** The PLOS ONE Staff

There are errors in Table 3. Many of the up arrows to indicate signal increases do not appear. Please see the correct Table 3 here. The publisher apologizes for the errors.

**Table 3:** Summarized histone modification levels during spermatogenesis and their aging-associates changes.

		pL(VIII)	L(X)	Z(XII)	P(I)	P(V)	P(VIII)	P(X)	M(XII)	R(I)	R(V)	R(VIII)	E(X)	Specific note
H3Kme2	Young	++++	+++++	++++	+++	+++	+	++	++++	+++++	+++++	++++	+++++	Accumulation on XY body
	Aged	↓↓**	↓↓**	↓↓**	↓↓**	↓↓**	↓↓**	↓↓**	↓↓**	↓↓**	↓↓**	↓↓**	↓↓**	
H3K4me3	Young	**-**	**-**	**-**	**-**	**-**	**-**	**-**	++++	**++++**	**+++++**	**++++**	**++++**	
	Aged	-	-	-	-	-	-	-	↓↓**	→	→	→	→	
H3K27ac	Young	+++++	+	+	+	++	++	++	+	++	++	++	+++++	Accumulation on XY body and sex chromosomes
	Aged	→	↓↓**	↓**	↑*	↓**	→	↓↓**	→	↑**	→	→	↑**	
H3K79me2	Young	**-**	**-**	**-**	**-**	**-**	**+**	**+**	**++**	**++**	**+++++**	**+++++**	**++++**	Accumulation on sex chromosomes
	Aged	**-**	**-**	**-**	**-**	**-**	**→**	↑**	**→**	**→**	**→**	↑*	↑↑**	
H3K79me3	Young	-	-	-	-	-	-	-	++++	-	++	++++	+++++	Accumulation on sex chromosomes
	Aged	-	-	-	-	-	-	-	↑↑**	-	→	↓*	↓**	
H3K9me3	Young	++	+++	+++	+++	+++	+	+	+	+	+	++	++	Accumulation on sex chromosomes and heterochromatin
	Aged	↓↓**	↓↓**	↓↓**	↓↓**	↓↓**	↓↓**	↓↓**	↓↓**	↓*	↑*	↓*	→	
H3K27me2	Young	-	+	+	+	++	++++	++	++	++	+++	++	++	Accumulation on heterochromatin
	Aged	-	↑↑**	↑↑**	↑↑**	↑↑**	↓*	↑↑**	↑↑**	↑**	↑↑**	→	→	
H3k27me3	Young	-	+++	++	++	++	++	++++	+++	+++	++++	+++++	+++++	Accumulation on heterochromatin
	Aged	-	↓↓**	↑↑**	↑↑**	↑↑**	↑↑**	↓↓**	→	↑↑**	↑↑**	→	→	

Accumulation levels of histone modifications that activate (red) and inhibit (blue) gene expression are shown. Each histone modification level in male germline cells derived from young mice is indicated as–(not detected), + (normalized intensity <0.4), ++ (0.4–0.6), +++ (0.6–0.8), ++++ (0.8–1.0), and +++++ (1.0<) (Actual values for intensity young male germline cells are shown in S1 Table). Arrows show histone modification levels of male germline cells derived from aged mice compared to that of young mice. Single up/down arrows indicate that a signal was increased/decreased by less than 20% of the mean intensity compared to the signal from young animals. Double up/down arrows indicate that the signal was increased/decreased by more than 20% compared to the signal from young animals. Right arrows indicate that there were no significant differences between young and aged animals (*p < 0.05, **p < 0.01). Specific localization or accumulation is described in the specific notes section.
