# Supplementary material for: Comprehensive histochemical profiles of histone modification in male germline cells during meiosis and spermiogenesis: Comparison of young and aged testes in mice
Source: PLoS One. 2020 Apr 8;15(4):e0230930. doi: 10.1371/journal.pone.0230930 (PMC7141650; doi:10.1371/journal.pone.0230930)
Supplement: S1 File — (DOCX) [file pone.0230930.s001.docx]

**DNA fluorescence *in situ* hybridization (DNA-FISH) followed by immunofluorescence staining**

Antigen retrieval and immunohistochemical staining were performed as described above. Rabbit anti-H3K79me3 (1:500, ab2621, Abcam) and mouse anti-α-Tubulin (T6199, Sigma-Aldrich) antibodies were used as primary antibodies. Alexa Fluor Plus 647 (1:500, A32728, Invitrogen) was used as secondary antibody, and slides were counterstained with DAPI. The sections were mounted with Vectashield (VECTOR LABORATORIES, INC.). After capturing images using a confocal laser-scanning microscope, glass coverslips were removed, and the sections were washed in PBS and immersed in Histo VT One (1:10, 06380-76, Nacalai Tesque) at 90°C for 25 minutes. After washing in PBS, the sections were immersed in 70% and 100% ethanol, dried in the air, and 10 μl of probes for X and Y chromosomes were applied (MXY-10, Chromosome Science Lab). The sections were covered with coverslips and heated at 80°C on a hot plate (HDB-1N, AS ONE) for 10 minutes. Then, after a 15-hour incubation in a humid chamber at 37°C, the sections were subjected to 2×standard saline citrate (SSC) (pH 4.0) washes, and the coverslips were removed. The sections were then incubated in 50% formamid-2×SSC at 37°C for 20 minutes and were subjected to 1×SSC wash for 15 minutes at room temperature. The sections were mounted with Vectashield. Images were captured using a confocal laser-scanning microscope.
