# Supplementary material for: Comprehensive histochemical profiles of histone modification in male germline cells during meiosis and spermiogenesis: Comparison of young and aged testes in mice
Source: PLoS One. 2020 Apr 8;15(4):e0230930. doi: 10.1371/journal.pone.0230930 (PMC7141650; doi:10.1371/journal.pone.0230930)
Supplement: S1 Table — (DOCX) [file pone.0230930.s002.docx]

|  | pL(VIII) | L (X) | Z(XII) | P(I) | P(V) | P(VIII) | P(X) | M(XII) | R(I) | R(V) | R(VIII) | E(X) |
| --- | --- | --- | --- | --- | --- | --- | --- | --- | --- | --- | --- | --- |
| H3K4me2 | 0.86644 | 1.09447 | 0.82303 | 0.78873 | 0.79064 | 0.29347 | 0.50901 | 0.99729 | 1.08986 | 1.53925 | 0.97614 | 1.02261 |
| H3K4me3 | n.d. | n.d. | n.d. | n.d. | n.d. | n.d. | n.d. | 0.85359 | 0.82838 | 1.01301 | 0.81813 | 0.82384 |
| H3K27ac | 1.37203 | 0.30389 | 0.29036 | 0.37848 | 0.52288 | 0.55344 | 0.56555 | 0.24605 | 0.45614 | 0.49629 | 0.51011 | 1.51854 |
| H3K79me2 | n.d. | n.d. | n.d. | n.d. | n.d. | 0.29954 | 0.38765 | 0.58133 | 0.53153 | 1.08888 | 1.12753 | 0.97633 |
| H3K79me3 | n.d. | n.d. | n.d. | n.d. | n.d. | n.d. | n.d. | 0.96961 | n.d. | 0.58526 | 0.97623 | 1.20561 |
| H3K9me3 | 0.54068 | 0.71888 | 0.68365 | 0.72539 | 0.63270 | 0.32366 | 0.21839 | 0.32427 | 0.27047 | 0.28329 | 0.51242 | 0.58995 |
| H3K27me2 | n.d. | 0.33776 | 0.26915 | 0.31625 | 0.45706 | 0.80556 | 0.58253 | 0.45566 | 0.50961 | 0.61101 | 0.66266 | 0.64939 |
| H3K27me3 | n.d. | 0.74808 | 0.46650 | 0.43003 | 0.49491 | 0.58854 | 0.96281 | 0.74516 | 0.63161 | 0.95806 | 1.004 | 1.40652 |
