# Supplementary figures and images for: Comprehensive histochemical profiles of histone modification in male germline cells during meiosis and spermiogenesis: Comparison of young and aged testes in mice
Source: PLoS One. 2020 Apr 8;15(4):e0230930. doi: 10.1371/journal.pone.0230930 (PMC7141650; doi:10.1371/journal.pone.0230930)

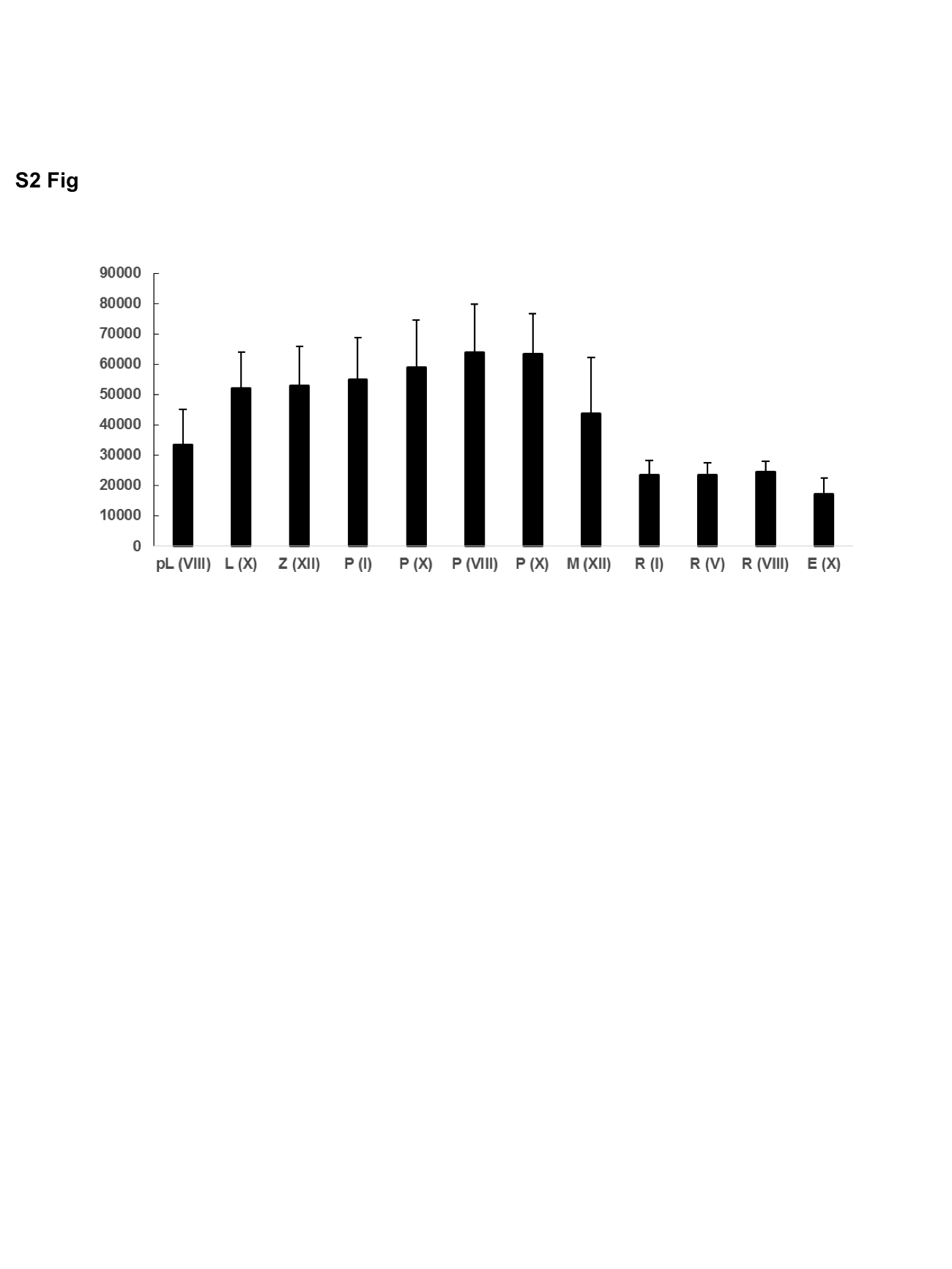

Supplement: S1 Fig — Integrated intensity of DAPI signal stained with histone modification signal was measured. Each parenthesis represents the stage of spermatogenesis (see Table 2.). Error bars show S.D. (TIFF) [file pone.0230930.s003.tiff]

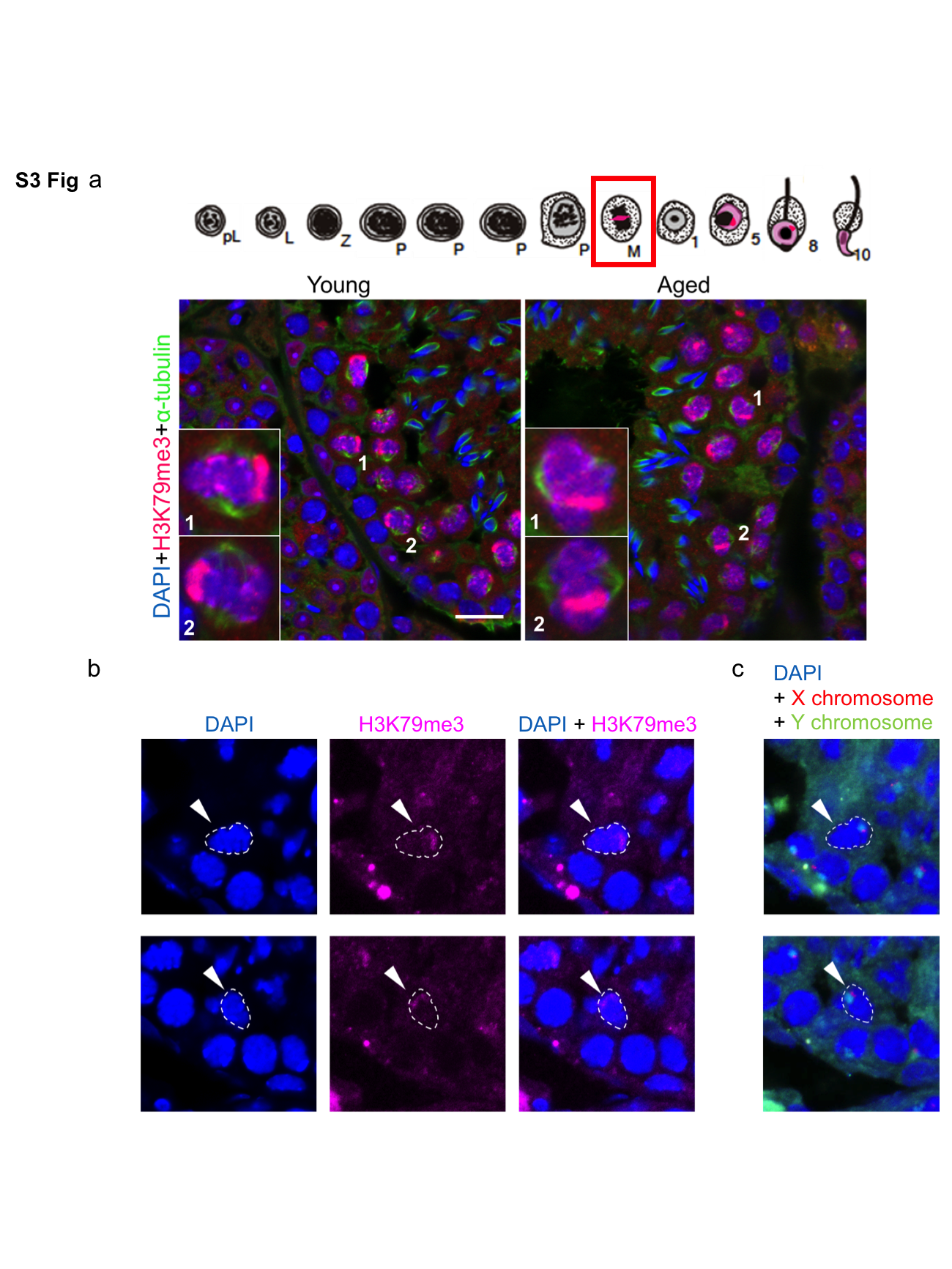

Supplement: S2 Fig — (a) An illustrated overview shows subcellular localization of H3K79me3 (magenta). Lower panels show representative confocal images of H3K79me3 (magenta) and α-tubulin (green) in M phase cells (red square in the summarized illustration) in young and aged testes. Nuclei were counterstained with DAPI (blue). Each magnified image of the cells is indicated by the number shown in the box. (b) Representative confocal images of H3K79me3 (magenta) in M phase cells. Nuclei were counterstained with DAPI (blue). M phase cells are indicated with dotted lines and arrowheads. (c) Representative images of FISH show X (red) and Y (green) chromosomes in M phase cells on the same section as (b). Nuclei were counterstained with Hoechst (blue). The same M phase cells as seen in (b) are indicated with dotted lines and arrowheads. (TIFF) [file pone.0230930.s004.tiff]
