# Supplementary material for: Comprehensive histochemical profiles of histone modification in male germline cells during meiosis and spermiogenesis: Comparison of young and aged testes in mice
Source: PLoS One. 2020 Apr 8;15(4):e0230930. doi: 10.1371/journal.pone.0230930 (PMC7141650; doi:10.1371/journal.pone.0230930)
Supplement: Attachment — Submitted filename: Response to Reviewers.docx [file pone.0230930.s006.docx]

Point-by-point responses for reviewer’s comments
**Reviewer #1:**

*This reviewer appreciate the efforts made by the authors to clarify their experimental procedure. Yet I still have major concerns about it.
1) The authors indicate that “Cells from multiple tubules were measured per testis, and four different young (3 months old) or aged (12 months old) mice were used, yielding 30-50 cells to be analyzed in total.”
which I understand to be 4 young males and 4 aged males: 50 cells in total in the 8 different animals or per animal ? because if it is 50 cells in total, this means, on average 6 cells per animal (per type of cells) which is clearly not enough.*

We apologize that our previous text was misleading. Since a certain type of cells, e.g., leptotene spermatocytes, in the testis were very small in number compared with other types of cells, we measured intensity of additional cells in the testis to obtain the same number of cells to be analyzed. We selected 50 cells for each cell type from four mice to make each box plot. Five to 15 cells (depends on cell types) from 2 or 3 tubules per mice were counted. Finally, we totally analyzed 50 cells for 12 cell types each in a group (for example, 600 cells in young and aged groups, respectively) (p. 10, lines 148-152), and confirmed that our results showed almost the same tendency as in the previous manuscript.

*2) I am questioning the quantification protocol. The reasoning behind using DAPI to normalize the antibody signal is unclear to me. Indeed, if there are problems of antibody accessibility or homogeneity within different regions of the slide/section during antibody incubation, they would not be picked up by DAPI staining, which is much more robust than antibody staining (and occurs at the end of the experimental procedure, not in parallel with antibody incubation).*

In our immunostaining procedures, all the slides for each histone modification staining were treated simultaneously under the same condition with a great care to ensure that the staining was uniform. Together with the antibody against each histone modification, we used the one against SCP3, a spermatocyte marker, to check the staining was uniform among tubules and to evaluate stages of the testis tubes. Since SCP3 showed robust staining as shown in Figs. 1 and 2, we assessed that our experimental procedures were suitable to obtain stable signals of each histone modification in male germline cells in both young and aged testes. The quality of the histone modification antibodies has been ensured in previous studies (see references listed in Table 1).

To reduce variable parameter, we used DAPI signal to normalize the histone modification signals in all the stage including elongating and elongated spermatids, which had already been agreed with Reviewer #3. It is difficult to normalize with pan H3 in elongating and elongated spermatids because histone-to-protamine replacement is taking place during these stages.

In this revision, we presented the total DAPI signal intensity at each spermatogenesis stage as supporting information (S2 Fig). Total intensity of DAPI signal dropped half between stage P(X) and R(I), where DNA amount in pachytene spermatocytes becomes half in the round spermatid (after meiosis), indicating that total DAPI signal intensity reflects changes in DNA amount. The DNA amount was variable in the preleptotene and metaphase spermatocytes, where DNA synthesis and chromosomal segregation occurs, respectively. From these results, we thought that the normalization by DAPI signal intensity would be reasonable as a stable standard for normalizing histone modification alteration throughout spermatogenesis (even after protamine replacement). Certainly, this method is semi-quantitative because a net amount of histone and the ratio of histone modification to the total histone is unknown. That is a reason that we had to present histone amount in aged mice as a relative intensity against to the histone amount in young mice. Changes in the net amount of histone during aging are also interesting but it requires further studies in the future. We added the explanation in Materials & Methods (p. 10, line 142-147).

The similar procedures have been applied in other studies (e.g., Ferguson et al., PLoS One, 2013.); the reference is added in the Materials & Methods (p. 10, line 140), and in References.

*3) Now that data as shown as box plots, one can see outliers as individual dots (sometimes more than 4 per group), and here I am confused because to me the correct way to measure statistical significance is to count (many) cells per individual, calculate the mean intensity, and plot the value obtained per male, and not individual cell values. But according to this figure, this is not how the authors perform their analysis.*

*Again, the topic of the study (impact of aging on histone PTM in male germ cells) is very interesting but to me the methodology (quantification method, sample size, statistical analyses) is not accurate to be able to conclude on whether or not there are significant changes.*

We appreciate this encouraging comment from the reviewer, and do hope our re-revision including re-analyses and reforming of data presentation would satisfy him/her.

For the style of data presentation, we did consider the style to show the values per male mice as proposed by this reviewer. We had discussed with the co-authors during our re-revision process, and we have finally adopted the current presentation style, showing scores per cells over the box plots, for the following reasons. First, our ultimate goal is to understand molecular mechanisms that underlie risks for neurodevelopmental diseases of children from the paternal side. We are wondering that altered histone modification in sperm cells could be one of such mechanisms. Since only one sperm cell will eventually be fertilized to develop a new individual, it would not be meaningful to discuss on the mean intensity of the cells per animal; it would mask individual changes in each sperm cell. Actually, the outliers were derived from different male mice. It could be possible that the cells/stages with several outliers could be critical timings vulnerable to alteration of histone modification during spermatogenesis.

Thus, we accordingly modified the text in Materials & Methods (p. 10, lines 147-148), Results (p. 23, lines 354-356), Figure Legends (p. 26, lines 405-406 and p. 28, lines 436-437), and Discussion (p. 30, lines 452-455 and p. 30, lines 460-463).

**Reviewer #2:**

*In this revised version, the authors provided the important and indispensable informations that were missing previously in paragraph of material and methods. Furthermore, changes introduced in every parts of the manuscript (title, summary, introduction, results, discussion and references) as well as a novel presentation of the experimental data (modification of figures, new table) have considerably improved this new version.

Nevertheless, to satisfy to the PLOS ONE criteria for publication, some modifications have to be introduced again in this revised version. Concerning the semi-quantitative analysis of the fluorescence intensity, explanations given on the experimental procedure, may allow a new presentation for the young males in Figure 1, with the indication of the mean of the fluorescence intensity by stage of differentiation. This would provide and complete the grayscale images of histone marks by a semi-quantitative data. Indeed, these experimental data are not accessible directly in the current presentation, because they are used indirectly through a normalization for the comparison between young and old males in Fig. 3. These data could be added in a modified Figure 1, or at least be accessible as supplementary data.*

We appreciate this comment from Reviewer #2. We added S1 Table to show the mean intensity of each type of the cells in the young group as Supporting Information (p. 48, line 849).

*In their reply, the authors failed to convince (reviewer 2) of the need to maintain a third paragraph of results (H3K79me3 on sex chromosome). As already mentioned, the co-localization of the H3K79me3 mark on XY chromosomes has been documented in metaphase and also suggested in round spermatids in the same publication (Ontoso D. et al., 2014). If the experimental approach developed by the authors (Immuno-FISH) does not justify, according to the authors themselves the novelty of the results presented, these data have to find their place in the supplemental data.
I (reviewer 2) owe you all my apologies for the errors introduced in the reference of publication previously cited for the protocol combining immunofluorescence and DNA-FISH. The right reference is: Donati C. et al, 2004 Journal of Histochemistry & Cytochemistry
PMID: 15385579
DOI: 10.1177/002215540405201009*

We agreed that there is no novelty in our data showing H3K79me3 on the sex chromosomes even though our Immuno-FISH method was newly developed. In the re-revised version, the data were shown as Supporting Information (S3 Fig, p. 25, lines 379-392 and p. 48, line 859-p. 50, line 889). *Finally, some indications for minor modifications are the following:
Page 10
- line 143, 30-50 cells … to be analyzed for each stage and each histone moiety.*
We changed the words as Reviewer #2 suggested (p.10, line 150).

*- line 146 : complete the citation for R and ggplot2 (check exemple above)
To cite ggplot2 in publications, please use:
- H. Wickham. ggplot2: Elegant Graphics for Data Analysis. Springer-Verlag New York, 2016.*

Thank you for suggesting us a suitable reference. We revised the citation for R and ggplot2 by referring the paper suggested (p. 11, lines 154-155).

*Page 11
line 169: complete the citation for JMP and the following link is suggested*
[*http://www.jmp.com/support/notes/35/282.html*](http://www.jmp.com/support/notes/35/282.html)

We checked the link and revised the citation for JMP (p. 11, lines 162-163). *Fig. 3
Line 389: correct the mistake in the legend because there is no arrowhead present in this figure.*

We appreciate Reviewer #2’s indication for the mistake that we inadvertently overlooked. We corrected the mistake in the legends of Fig 3 as well as of Fig 4 (p. 26, lines 402-403 and p. 28, lines 433-434, respectively).
